# Altered Gut Microbiota and Its Metabolites in Hypertension of Developmental Origins: Exploring Differences between Fructose and Antibiotics Exposure

**DOI:** 10.3390/ijms22052674

**Published:** 2021-03-06

**Authors:** Chien-Ning Hsu, Julie Y. H. Chan, Kay L. H. Wu, Hong-Ren Yu, Wei-Chia Lee, Chih-Yao Hou, You-Lin Tain

**Affiliations:** 1 Department of Pharmacy, Kaohsiung Chang Gung Memorial Hospital, Kaohsiung 833, Taiwan; cnhsu@cgmh.org.tw; 2 School of Pharmacy, Kaohsiung Medical University, Kaohsiung 807, Taiwan; 3 Institute for Translational Research in Biomedicine, Kaohsiung Chang Gung Memorial Hospital and Chang Gung University College of Medicine, Kaohsiung 833, Taiwan; jchan@cgmh.org.tw (J.Y.H.C.); wlh0701@yahoo.com.tw (K.L.H.W.); 4 Department of Pediatrics, Kaohsiung Chang Gung Memorial Hospital, Chang Gung University College of Medicine, Kaohsiung 833, Taiwan; yuu2002@cgmh.org.tw; 5 Department of Urology, Kaohsiung Chang Gung Memorial Hospital, Chang Gung University College of Medicine, Kaohsiung 833, Taiwan; dinor666@ms32.hinet.net; 6 Department of Seafood Science, National Kaohsiung University of Science and Technology, Kaohsiung 811, Taiwan; chihyaohou@webmail.nkmu.edu.tw

**Keywords:** developmental origins of health and disease (DOHaD), fructose, gut microbiota, hypertension, minocycline, nitric oxide, renin–angiotensin system, short chain fatty acid

## Abstract

Gut microbiota-derived metabolites, in particular short chain fatty acids (SCFAs) and their receptors, are linked to hypertension. Fructose and antibiotics are commonly used worldwide, and they have a negative impact on the gut microbiota. Our previous study revealed that maternal high-fructose (HF) diet-induced hypertension in adult offspring is relevant to altered gut microbiome and its metabolites. We, therefore, intended to examine whether minocycline administration during pregnancy and lactation may further affect blood pressure (BP) programmed by maternal HF intake via mediating gut microbiota and SCFAs. Pregnant Sprague-Dawley rats received a normal diet or diet containing 60% fructose throughout pregnancy and lactation periods. Additionally, pregnant dams received minocycline (50 mg/kg/day) via oral gavage or a vehicle during pregnancy and lactation periods. Four groups of male offspring were studied (*n* = 8 per group): normal diet (ND), high-fructose diet (HF), normal diet + minocycline (NDM), and HF + minocycline (HFM). Male offspring were killed at 12 weeks of age. We observed that the HF diet and minocycline administration, both individually and together, causes the elevation of BP in adult male offspring, while there is no synergistic effect between them. Four groups displayed distinct enterotypes. Minocycline treatment leads to an increase in the F/B ratio, but decreased abundance of genera *Lactobacillus*, *Ruminococcus*, and *Odoribacter*. Additionally, minocycline treatment decreases plasma acetic acid and butyric acid levels. Hypertension programmed by maternal HF diet plus minocycline exposure is related to the increased expression of several SCFA receptors. Moreover, minocycline- and HF-induced hypertension, individually or together, is associated with the aberrant activation of the renin–angiotensin system (RAS). Conclusively, our results provide a new insight into the support of gut microbiota and its metabolite SCAFs in the developmental programming of hypertension and cast new light on the role of RAS in this process, which will help prevent hypertension programmed by maternal high-fructose and antibiotic exposure.

## 1. Introduction

Hypertension is considered to be an increasingly common driver of the epidemic of non-communicable disease around the world [1]. In 2015, the WHO indicated that 1 in 4 men and 1 in 5 women had hypertension [2]. Accumulative evidence demonstrates that adverse in utero environments can increase the risk of developing hypertension in later life [3,4,5]. Today, this theory is called the Developmental Origins of Health and Disease (DOHaD) [6]. On the other hand, programming processes might be reversed by shifting the therapeutic interventions from the adulthood to fetal stage, namely, reprogramming, to prevent the transition from non-disease to disease in later life [7].

Reshaping microbiota during the critical phases of development is reported to have a lifelong influence on offspring health outcomes [8]. As reviewed elsewhere, such programming is beneficial or harmful depending on the circumstances [9,10]. A variety of nutritional insults during pregnancy can cause programming, leading to adult-onset diseases. Conversely, maternal nutritional interventions can serve as reprogramming strategies to protect offspring against many diseases in adulthood, including hypertension [10]. Maternal diet has been shown to impact the maternal and infant gut microbiome [11]. Offspring from pregnancies where maternal diets are high in fructose are at greater risk for adverse health outcomes [12]. As we previously reported, offspring born to dams exposed to a 60% high-fructose diet develop hypertension in adulthood, which is associated with reshaping microbiota and alterations of its metabolite short chain fatty acids (SCFAs) [13,14].

Medication use in pregnancy can also affect gut microbiota. Alterations of gut microbiota by antibiotics had beneficial or detrimental roles in the context of hypertension, depending on the types of antibiotics and genotypes [15]. Although antibiotics are effective and potentially life saving for pregnant women with bacterial infections, their uses can alter the mother’s and, therefore, their offspring’s microbiome. Data obtained from both human and animal studies indicate that the intrapartum administration of antibiotics affects the development of gut microbiota in offspring [16,17].

The gut microbiota contributes to the regulation of blood pressure (BP) via its metabolites to stimulate the enteric afferent sensory fibers or affect the target organs responsible for BP regulation, such the kidney [18,19,20]. However, most studies currently reporting links between gut microbiome and BP are conducted in established hypertension, rather than hypertension of developmental origins [18,19,20]. Only a few studies demonstrated that the modulation of gut microbiota by probiotics or prebiotics in pregnancy and lactation can prevent adult offspring against hypertension programmed by maternal nutritional insults [14,21]. Whether such a reshaping of microbiota programmed by antibiotics administration during pregnancy and lactation impacts offspring BP remains unknown. In addition to microbiota dysbiosis, nitric oxide (NO) deficiency and the aberrant renin–angiotensin system (RAS) have been associated with hypertension of developmental origins [5,7,22]. Our prior work implicated NO and RAS pathways in a maternal HF diet plus a post-weaning high-fat diet model [23].

Minocycline, a tetracycline, is active against both Gram-positive and Gram-negative bacteria [24]. Minocycline has been reported to reshape dysbiotic gut microbiota and reduce BP in spontaneously hypertensive rats (SHR), a commonly used model of hypertension [18,25]. Nevertheless, whether the perinatal administration of minocycline affects offspring’s BP is largely unknown. The aim of this study was to test whether minocycline administration in pregnancy and lactation attenuate or aggravate hypertension programmed by maternal HF intake in adult offspring related to changes in the gut microbiota composition, SCFAs and their receptors, NO and the RAS.

## 2. Results

### 2.1. Renal Outcome and BP

We examined four groups of male offspring born to mother rats that received a normal diet (ND), a high-fructose diet (HF), a normal diet and minocycline administration (NDM), and a HF diet and minocycline administration (HFM). Table 1 shows that three offspring died in the HFM group: one died at 8 weeks old, and the others died at 10 weeks old. All of them displayed irritable behavior during BP measurement and died shortly after returning back to cages. The maternal HF diet caused a higher body weight (BW) (P_HF_ = 0.004) and kidney weight (P_HF_ = 0.018) in the HF group compared to those in the ND group, while minocycline only had an effect on BW (P_M_ = 0.004). There was a significant effect of minocycline on the kidney weight-to-BW ratio (P_M_ = 0.016); minocycline caused a higher ratio in the NDM and HFM group compared to the ND and HF groups, respectively.

**Figure 1:**
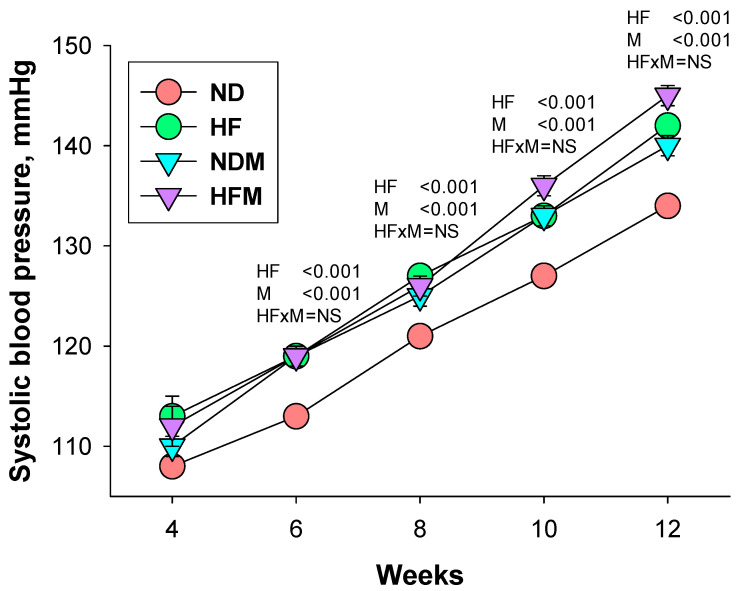
Effect of high-fructose diet (HF) and minocycline administration (M) on systolic blood pressure in offspring from 3 to 12 weeks of age. HF × M = the interaction between high-fructose diet and minocycline.

Figure 1 shows measurements of longitudinal systolic blood pressure (SBP). High-fructose diet and minocycline administration, individually or together, caused an elevation of SBP from six to 12 weeks (P_HF_ < 0.001 and P_M_ < 0.001). At 12 weeks of age, offspring in the HF group had a higher SBP and mean arterial pressure (MAP) compared to ND offspring. Likewise, minocycline administration significantly increased SBP, diastolic BP (DBP), and MAP in the NDM group. Although combined HF and minocycline increased SBP, DBP, and MAP in the HFM group, there was no interaction between HF and minocycline treatment. Our data demonstrated that there was no synergistic interaction between the high-fructose diet and minocycline administration on BPs, which indicated that minocycline did not intensify or mitigate maternal HF-induced hypertension in adult male offspring. Additionally, we observed that minocycline caused a lower creatinine level (P_M_ = 0.002) and higher clearance of creatinine (CCr; P_M_ = 0.001) in the NDM and HFM group compared to the respective ND and HF groups.

### 2.2. SCFA Levels and SCFA Receptors

We first investigated whether HF and minocycline exposure affected SCFA levels in the feces and plasma. As shown in Table 2, maternal HF intake displayed a significant effect on levels of isobutyric acid, isovaleric acid, and valeric acid in feces and acetic acid in the plasma. Plasma levels of acetic acid and butyric acid were reduced in the minocycline-exposed offspring rats. However, both HF and minocycline had negligible effects on the plasma levels of acetic acid, propionic acid, and butyric acid.

**Figure 2:**
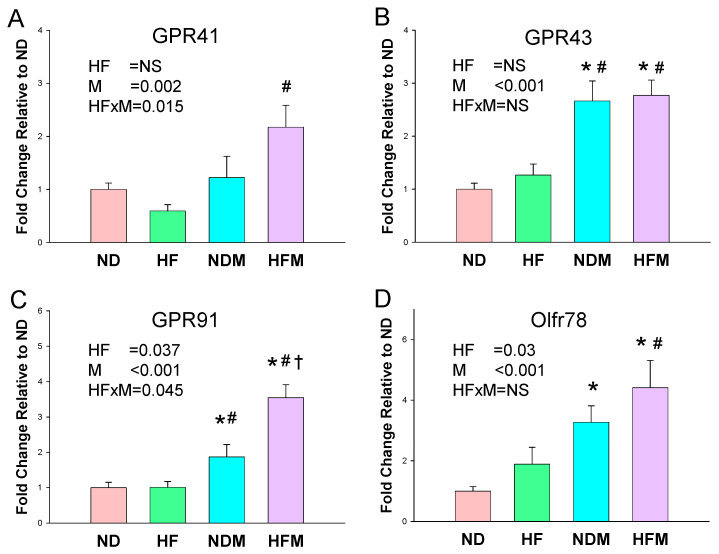
Effect of high-fructose diet (HF) and minocycline administration (M) on short chain fatty acid (SCFA) receptors in offspring kidneys. The mRNA expression of SCFA receptor (**A**) G protein-coupled receptor 41 (GPR41), (**B**) GPR43, (**C**) GPR91, and (**D**) olfactory receptor 78 (Oflr78); HF × M = the interaction between high-fructose diet and minocycline. NS = not significant; HF × M = the interaction between high-fructose diet and minocycline. * *p* < 0.05 vs. ND; # *p* < 0.05 vs. HF; † *p* < 0.05 vs. NDM.

According to our data (Figure 2), HF affected SCFA receptors slightly in the renal mRNA expression of the G protein-coupled receptor 91 (GPR91) and olfactory receptor 78 (Oflr78). However, minocycline had a significant effect on GPR41, GPR43, GPR91, and Oflr78 levels in offspring kidneys. Almost all SCFA receptors were augmented by maternal minocycline treatment. Both HF and minocycline had synergistic effects on the mRNA expression of GPR41 and GPR 91.

### 2.3. Gut Microbiota Composition

**Figure 3:**
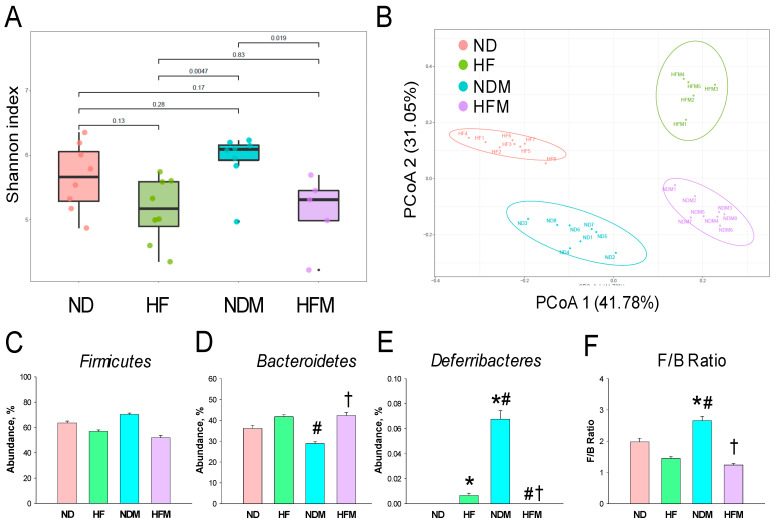
Effect of high-fructose diet (HF) and minocycline administration (M) on the gut microbiome in male offspring. (**A**) Shannon’s α-diversity indexes. (**B**) β-diversity using the Principal Coordinate Analysis (PCoA). Relative abundance of the phylum (**C**) *Firmicutes*, (**D**) *Bacteroidetes*, and (**E**) *Deferribacteres*. (**F**) The *Firmicutes* to *Bacteroidetes* (F/B) ratio. Data are shown as means ± SEM; N = 5–8/group. * *p* < 0.05 vs. ND; # *p* < 0.05 vs. HF; † *p* < 0.05 vs. NDM.

We next examined microbiome communities among the four groups. Microbiome diversity was assessed by measuring within (i.e., α-diversity) and between community/sample (i.e., β-diversity) diversities [26]. Maternal HF intake had no effect on α-diversity, represented by Shannon (Figure 3A) and Simpson indexes that relate to microbial species richness and evenness. However, minocycline administration caused a higher α-diversity for the Shannon and Simpson indexes (both *p* < 0.05) in the NDM group than that in the HF and HFM groups. We determined β-diversity by using PCoA plots. The unweighted UniFrac distance matrices were used to construct PCoA plots. This is the percentage variance explained by each axis: PCoA 1 = 41.78% and PCoA 2 = 31.05%. As shown in Figure 3B, the PCoA plots showed significant clustering according to study group, indicating that the gut microbiota structure in the ND group was distinctly altered by HF, minocycline, and combined HF and minocycline exposure. These data indicate that HF diet and minocycline treatment, individually or together, significantly changed gut microbiota composition. At the phylum level, the major bacteria phyla found are *Firmicutes, Bacteroidetes, Deferribacteres, Actinobacteria, Proteobacteria*, and *Verrucomicrobia* in 12-week-old offspring. The abundance of the phylum *Firmicutes* was not different among the four groups (Figure 3C). The abundance of phylum *Bacteroidetes* was higher in the HFM group compared to the NDM group (Figure 3D). Additionally, the HF and NDM groups had a higher phylum *Deferribacteres* abundance compared to the ND and HFM groups (Figure 3E). The *Firmicutes* to *Bacteroidetes* (F/B) ratio has been considered a characteristic of hypertension [18]. We observed that the NDM group displayed the highest F/B ratio among the four groups (Figure 3F).

**Figure 4:**
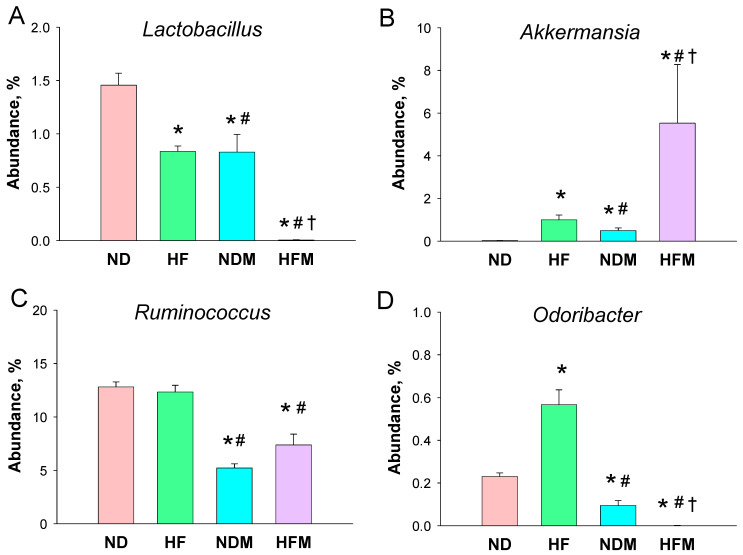
Effect of high-fructose diet (HF) and minocycline administration (M) on the gut microbiome in male offspring. Relative abundance of the genera (**A**) *Lactobacillus,* (**B**) *Akkermansia,* (**C**) *Ruminococcus*, and (**D**) *Odoribacter*. Data are shown as means ± SEM; *N* = 5–8/group. * *p* < 0.05 vs. ND; # *p* < 0.05 vs. HF; † *p* < 0.05 vs. NDM.

As a result, *Lactobacillus* of the genus-level was significantly reduced by HF diet and minocycline administration, both individually and together (Figure 4A). The maternal HF diet and minocycline treatment caused a notable increase in the abundance of genus *Akkermansia* in the HF and NDM groups, with a highest abundance in the HFM group (Figure 4B). Additionally, minocycline reduced the abundance of the genus *Ruminococcus* in the NDM and HFM groups compared to the other two groups (Figure 4C). Moreover, the abundance of genus *Odoribacter* was higher in the HF group than that in the ND group, which was reduced by minocycline treatment in the NDM and HFM group (Figure 4D).

**Figure 5:**
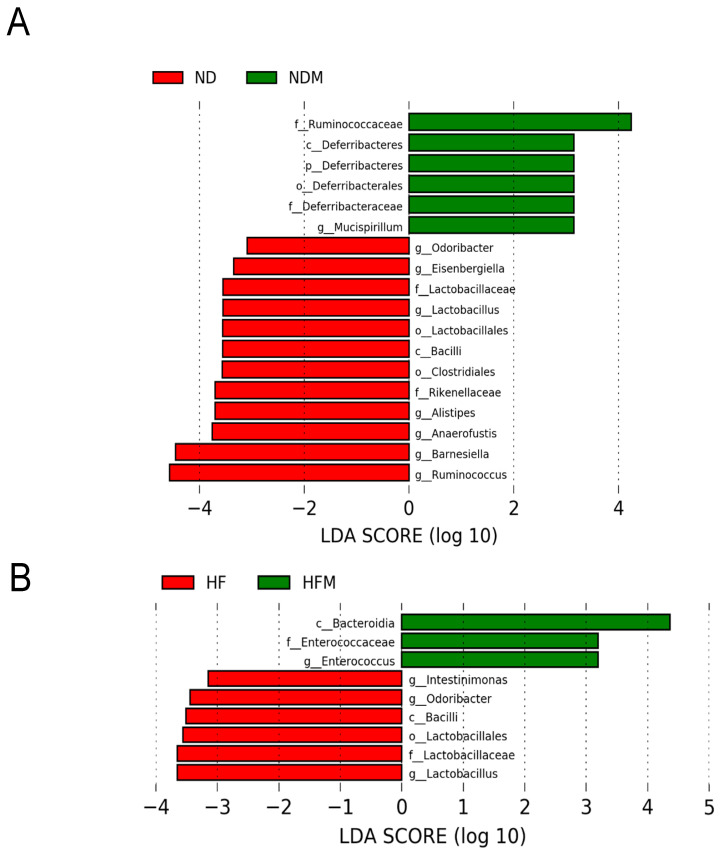
Effect of high-fructose diet (HF) and minocycline administration (M) on the gut microbiome in male 12-week-old male offspring. Linear discriminant analysis effect size (LEfSe) was applied for biomarker discovery in metagenomic data. Most enriched and depleted bacterial taxa in the (**A**) ND (red) versus NDM group (green) and (**B**) HF (red) versus HFM group (green) are shown. Different taxonomic levels of bacteria are given reaching from phylum down to genus-level. Each name is preceded by a letter giving the rank: p = phylum, c = class, o = order, f = family, g = genus. The threshold on the linear discriminant was set to 3.

Figure 5 illustrates statistically significant biomarkers between groups, which were identified by the linear discriminant analysis effect size (LEfSe) algorithm, a high-dimensional biomarker discovery method. We applied LEfSe analysis and found that there was a lower abundance of genera *Lactobacillus, Ruminococcus*, and *Odoribacter* in the NDM group vs. the ND group (Figure 5A). Similarly, minocycline administration caused a lower abundance of genera *Lactobacillus* and *Odoribacter* in the HFM group than that in the HF group (Figure 5B). Furthermore, the LEfSe analysis identified that the abundance of class *Bacilli*, a major class related to butyrate synthesis [24], was significantly reduced in the minocycline-treated NDM and HFM groups (Figure 5A,B).

### 2.4. NO-Related Parameters

As NO deficiency is involved in the developmental programming of hypertension [22], we next analyzed the NO pathway (Table 3). The plasma levels of l-citrulline (the precursor of l-arginine) and l-arginine (the substrate for NO synthase) were higher in the NDM group compared to the ND group, whereas these increases were reduced in the HFM group. However, plasma levels of asymmetric and symmetric dimethylarginine (ADMA and SDMA, both are inhibitors of NO synthase), and the l-arginine-to-ADMA ratio (an index of NO bioavailability), were comparable among the four groups.

### 2.5. Renin–Angiotensin System

**Figure 6:**
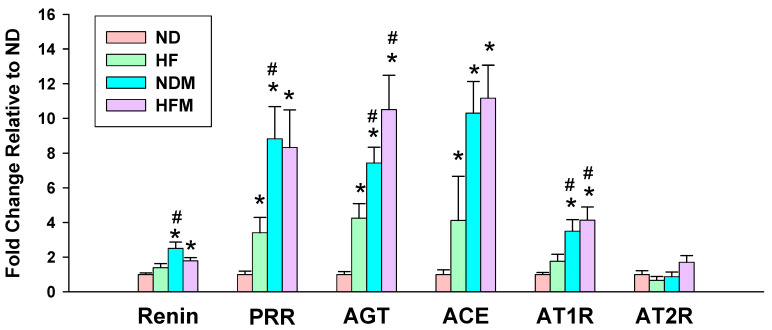
Effect of high-fructose diet (HF) and minocycline administration (M) on the renin–angiotensin system in 12-week-old male offspring kidneys. PRR = (Pro)renin receptor; AGT = angiotensinogen; ACE = angiotensin-converting enzyme; AT1R = angiotensin II type 1 receptor; AT2R = angiotensin II type 2 receptor; Data are shown as means ± SEM; *N* = 5–8/group; * *p* < 0.05 vs. ND; # *p* < 0.05 vs. HF.

Another important mechanism underlying hypertension of developmental origins is aberrant RAS [5,22]. We further determined the mRNA expression of the RAS components in offspring kidneys (Figure 6). There was a higher renal mRNA expression of (pro)renin receptor (PRR), angiotensinogen (AGT), and angiotensin-converting enzyme (ACE) in the HF group compared with those in ND rats. Minocycline treatment significantly increased mRNA levels of renin, PRR, AGT, ACE, and angiotensin II type 1 receptor (AT1R) in the NDM group vs. ND group. Similarly, minocycline treatment caused higher mRNA levels of AGT and AT1R in HFM offspring compared to HF offspring. However, AT2R expression did not differ among the four groups.

## 3. Discussion

The most significant findings of the current study are: (1) high-fructose diet and minocycline administration in pregnancy and lactation, both individually and together, cause a rise in BP in adult male offspring, while there is no synergistic effect between them; (2) maternal HF and minocycline treatment differentially shape gut microbiota profile, leading to the distinct enterotypes of four groups; (3) minocycline treatment leads to an increase in the F/B ratio, but decreases in the genera *Lactobacillus,*, *Ruminococcus*, and *Odoribacter* abundance; (4) maternal minocycline administration induces offspring’s hypertension coinciding with decreases in plasma acetic acid and butyric acid concentrations; (4) hypertension programmed by maternal HF diet plus minocycline exposure is related to the increase in the renal mRNA expression of GPR43, GPR91, and Oflr78; (5) minocycline-induced programmed hypertension is associated with the activation of the classical RAS with the increased expression of renin, PRR, AGT, ACE, and AT1R; and (6) maternal HF-induced hypertension in adult offspring is also relevant to aberrant RAS, represented by increases in PRR, AGT, and ACE. Taken together, these data may provide new insights into the pathogenesis of hypertension programmed by maternal HF or minocycline exposure, and we can expect the development of novel protective strategies against hypertension of developmental origins.

To the best of our knowledge, the present study is the first to show that maternal minocycline treatment causes the elevation of BP in adult male offspring. Our finding conflicts with prior studies showing that minocycline is able to reduce BP in animals with established hypertension [18,25]. It is possible that the mechanisms through which minocycline directly mediates BP in established hypertension are different from those involved in hypertension of developmental origins. Similar to previous studies [15,23], the maternal HF diet induced hypertension in adult offspring. However, combined HF and minocycline exposure brought about a negligible synergistic effect on offspring’s BPs. The present study is inconsistent with previous reports showing that different early-life insults may induce programmed hypertension synergistically [23,27]. However, HF intake and minocycline individually had no effect on mortality, but both of them together led to reduced offspring survival. Our data imply that the programming effect of different insults might be overlapped or independent, causing complex patterns that are difficult to decipher.

We observed that maternal HF diet and minocycline exposure, both individually and together, shape offspring gut microbiome distinctly. Minocycline treatment causes an increase in α-diversity in the NDM group compared to the HF and HFM groups, although three groups displayed similar degrees of high BP. Although previous studies have shown that a higher α-diversity is more beneficial and its decrease is related to hypertension [18,19], our results suggest that reduced microbial richness and diversity might not be a major determinant of mechanisms underlying hypertension of developmental origins in this model. Additionally, a lower α-diversity is also associated with obesity [28]. Noteworthily, minocycline treatment-increased microbial α-diversity is associated with decreases in BW but not BPs in adult offspring exposed to maternal HF intake. Our findings imply that the underlying mechanisms behind maternal HF intake-induced obesity and hypertension might be disassociated, and obesity is more closely associated with the loss of microbial diversity. Interestingly, minocycline and HF intake individually caused a trend for an increased abundance of genus *Akkermansia*, with both of them together having the largest effect. Since minocycline treatment augmented the *Akkermansia* abundance and attenuated HF-induced obesity and *Akkermansia* plays a decisive role in reducing the risk of obesity [29], additional study is warranted to clarify whether the protective effect of minocycline on obesity is directly attributed to its regulation on the *Akkermansia*.

Minocycline treatment led to the highest F/B ratio in the NDM group, which corresponded to elevated BPs. Our data support the notion of an increased F/B ratio related to hypertension as previously published [18,19]. Additionally, minocycline-induced programmed hypertension is associated with the remodeling of gut microbiota, with a particular reduction in the genera *Lactobacillus, Ruminococcus*, and *Odoribacter*. *Lactobacillus* spp. are microorganisms commonly used as probiotics to help enhance gut health [30]. The data in this work demonstrated that minocycline treatment not only reduced genus *Lactobacillus* abundance in the NDM offspring but also further exacerbated the decreases in *Lactobacillus* abundance programmed by maternal HF intake in the HFM group. This finding is unsurprising in view of prior reports revealing the positive effects of *Lactobacillus* on BP [13,21,31]. According to our data, minocycline programmed hypertension in adult offspring coincided with a decrease in genera *Ruminococcus* and *Odoribacter* abundance, which was in agreement with previous studies demonstrating that their abundance was deficient in individuals with hypertension [32,33].

Similar to our previous findings [14], maternal HF-induced hypertension is associated with increased plasma levels of acetic acid. The results of the present study go beyond prior reports, demonstrating that hypertension induced by maternal HF intake is also related to increased fecal levels of isobutyric acid, isovaleric acid, and valeric acid. Given that acetic acid has a vasodilatory effect and acetate supplementation protects against hypertension programmed by maternal HF consumption [14], and that plasma levels of isobutyric acid and isovaleric acid are negatively correlated with BP [34], the increases in these SCFAs might be a counterbalancing mechanism but not a cause of HF-induced programmed hypertension.

Unlike HF exposure, minocycline treatment reduced the plasma levels of acetic acid and butyric acid, as well as upregulated several SCFA receptors. Reduced acetic acid levels might be due to minocycline treatment that decreased acetate-producing bacteria, such as *Lactobacillus*. Likewise, minocycline-induced reduction in butyric acid is possibly related to its inhibition of the growth of butyrate-producing microbes, such as class *Bacilli* and genus *Ruminococcus* [35]. Additionally, both acetic acid and butyric acid are ligands for GPR41, which has a BP-lowering effect [36]. Conversely, the vasodilatory action of GRP41 can be counteracted by GPR43 and Olfr78, which are reported to mediate vasoconstriction in hypertension [37]. Of note is that minocycline treatment significantly increased SCFA receptor GPR43 and Oflr78 levels in the NDM and HFM groups. Thus, the programming effects of minocycline-induced hypertension might be attributed to its regulation on certain SCFAs and their receptors and the shift of the vasoconstriction-vasodilation balance in favor of vasoconstriction.

Another mechanism underlying hypertension of developmental origins could be due to the aberrant activation of the RAS. Findings of the current study are in agreement with previous reports showing that the aberrant activation of the RAS is related to hypertension programmed by maternal HF consumption [38,39]. We found that minocycline-induced programmed hypertension is associated with the activation of the classical ACE-angiotensin II (Ang II)-AT1R axis. Additionally, minocycline increased renin and PRR expression. Binding of renin to the PRR can activate Ang II-dependent and -independent pathways, leading to hypertension [40]. A previous report demonstrated that Olfr78 elevates BP via the mediation of renin secretion [41]. Given that minocycline treatment-induced programmed hypertension coincided with increased Oflr78 and renin expression, further research is needed to clarify whether the interplay between SCFA receptors and RAS could be a potential target for preventing hypertension of developmental origin.

In addition to the RAS, we focused on the NO pathway due to its roles in the control of BP and kidney development [42]. According to our data, minocycline treatment increased plasma L-citrulline and L-arginine levels, while these increases were reduced by HF exposure. Despite this, we noted that ADMA, SDMA, and L-arginine-to-ADMA ratio were not different among the four groups. Therefore, the overall NO pathway might not be a major factor contributing to the development of hypertension in this model. Nevertheless, the implications of minocycline-induced increases in L-citrulline and L-arginine deserve further clarification.

In the current study, we tested the hypothesis that the reshaping of gut microbiome in early critical developmental phases affects BP in later life and showed that the administration of minocycline during pregnancy and lactation increased offspring’s BP, which coincided with the lowering of acetate and butyrate, upregulating SCFA receptors, and aberrantly activating the RAS. Although this is a novel and exciting finding, the study still has some limitations. First, this is a male-only study. We previously reported that maternal HF-induced hypertension in a sex-dependent manner [43]. Whether there exists a sex difference in the programming effects of minocycline treatment deserves further elucidation. Second, we fully understand that the above-mentioned mechanisms in the current study might not cover the whole picture of the programming effects of minocycline treatment and HF intake, individually or together, on hypertension of developmental programming. A thorough examination of other mechanisms underlying hypertension of developmental programming is worthy of further study. Third, we analyzed the gut microbiota profile and SCAF levels only in 12-week-old offspring; the observed changes might be a consequence of programmed hypertension. Studying gut microbiota and SCFA levels in 3-week-old offspring after weaning and in dams might provide more details on how minocycline influences the gut microbiome. Last, the observations presented in our study are useful for indicating that minocycline and HF intake has detrimental effects on offspring BP, but are only limited to testing in this model. Obviously, additional studies are required in other animal models of developmental programming and in humans before this is translated into a clinical reality.

## 4. Materials and Methods

### 4.1. Animals and Experimental Design

The study was conducted according to the guidelines of the Guide for the Care and Use of Laboratory Animals of the National Institutes of Health, and approved by the Institutional Animal Ethics Committee of Chang Gung Memorial Hospital (Permit number: 2019011001; approval date: 31 January 2019). Virgin Sprague Dawley (SD) rats were purchased from BioLASCO Taiwan Co. Ltd. (Taipei, Taiwan). Rats were housed in an AAALAC-International accredited animal facility. Breeding rats were housed together overnight. Mating was confirmed by the presence of a copulatory plug. Pregnant rats were randomly divided into 4 groups and fed as follows during pregnancy and lactation periods: (1) ND, normal diet and vehicle; (2) HF, diet containing 60% fructose and vehicle; (3) NDM, normal diet plus minocycline administration (50 mg/kg/day) via oral gavage; and (4) HFM, diet containing 60% fructose plus minocycline administration (50 mg/kg/day) via oral gavage. The doses of fructose and minocycline used here were based on previous studies conducted in rodents, which were able to affect BP [13,18]. In order to better control the quality of milk and standardize the maternal care, the litters were down-sized to eight pups per mother after birth. Only male offspring from litters were used in subsequent experiments, as males are more prone to hypertension at a younger age [44].

BP was measured in rats under conscious conditions at age of 4, 6, 8, 10, and 12 weeks using an indirect tail-cuff method (CODA, Kent Scientific Corporation, Torrington, CT, USA). To ensure accuracy and reproducibility, the rats were allowed to adapt to restraint and tail-cuff inflation for 1 week prior to the experiment. BP measurements were taken between 13:00 and 17:00 each day on a blinded basis by the same experienced research assistant, as described previously [13,14]. All rats were sacrificed at 12 weeks of age. Fresh feces samples were collected, frozen, and stored at −20 °C until extraction. Blood samples were collected in heparinized tubes. The perfused kidneys were harvested, divided into cortex and medulla, and placed in a −80 °C freezer until analysis.

### 4.2. Gas Chromatography-Mass Spectrometry (GC-MS)

Gas chromatography-mass spectrometry (7890B, Agilent Technologies Wilmington, DE, USA) equipped with an automated sampler was applied to determine plasma and fecal levels of acetate, propionate, isobutyric acid, butyric acid, isovaleric acid, and valeric acid. According to our validated protocol [45], chromatographic separation was achieved using a DB-FFAP column (30 cm × 0.25 mm, 0.25 µm; Agilent Technologies, Wilmington, DE, USA). An injection volume of 1 μL with split ratio 5:1 was performed at 240 °C. 2-ethylbutiric acid was used as the internal standard. Fecal concentrations of SCFAs were represented as mM/g feces.

### 4.3. Quantitative Real-Time Polymerase Chain Reaction (qPCR)

RNA was extracted from the kidney cortex of each rat and analyzed by qPCR separately according to the previously described procedures [13,14]. The complementary DNA (cDNA) product was synthesized using a MMLV Reverse Transcriptase (Invitrogen, Carlsbad, CA, USA). Two-step quantitative real-time PCR was performed using the Quantitect SYBR Green PCR Reagents kit (Qiagen, Valencia, CA, USA) and the iCycler iQ Real-Time PCR Detection System (Bio-Rad, Hercules, CA, USA). We analyzed the following SCFA receptors, including G protein-coupled receptor 41 (GPR41), GPR43, GPR91, and olfactory receptor 78 (Oflr78). Additionally, several components of the RAS were analyzed, including angiotensinogen, renin, prorenin receptor (PRR), angiotensin converting enzyme (ACE), and angiotensin II type 1 receptor (AT1R). We used the R18S reference gene as the internal control because its expression level was constant across all the test samples. Each sample was run in duplicate. Primers were designed using GeneTool Software (BioTools, Edmonton, AB, Canada). Primer efficiency between 1.8 and 2.2 was acceptable. Table 4 shows the primer sequences of qPCR. We used the comparative threshold cycle (Ct) method to quantify the relative gene expression. The fold-increase in the experimental sample, relative to the control, was calculated using formula 2^-ΔΔCt^.

### 4.4. Metagenomics Analysis of Gut Microbiota

Metagenomic DNA was extracted from frozen fecal samples using a fecal DNA isolation kit according to the manufacturer’s instructions (EasyPrep Stool Genomic DNA Kit, Biotools Co., Ltd., New Taipei City, Taiwan), as described previously [21,45]. The frozen storage time of feces was less than 24 h. Amplicons were prepared according to the 16S Metagenomics Sequencing Library Preparation protocol (Illumina, San Diego, CA, USA), and sequenced with the Illumina MiSeq platform (Illumina, San Diego, CA, USA) at the Genomic and Proteomic Core Laboratory, Kaohsiung Chang Gung Memorial Hospital (Kaohsiung, Taiwan). Next generation sequencing data were analyzed with the Microbial Genomics Module of CLC Genomics Workbench 9.5.4 (Qiagen, Stockach, Germany). Illumina sequence data were processed using QIIME version 1.9.1. The sequences were clustered into operational taxonomic units (OTUs) at 97% similarity using the USEARCH algorithm. The phylogenetic relationships were constructed based on a representative sequence alignment with Fast-Tree. The α-diversity was quantified as observed OTU counts and Shannon and Simpson diversity indexes. We also compared the patterns of β-diversity for microbial communities by PCoA. To further determine the significantly differential taxa, LEfSe was applied to compare samples between groups. The threshold of the linear discriminant was set to 3.

### 4.5. High Performance Liquid Chromatography

According to a protocol validated in our lab [45], plasma levels of L-arginine (the substrate for NO synthase), L-citrulline (the precursor of L-arginine), ADMA, and SDMA were determined by HPLC (HP series 1100, Agilent Technologies, Inc., Santa Clara, CA, USA) with the O-phthalaldehyde/3-mercaptopropionic acid (OPA/3MPA) derivatization reagent.

### 4.6. Statistical Analysis

Data are given as the mean ± the standard error of the mean (SEM). A *p*-value < 0.05 was considered statistically significant for all tests. Statistical analysis was performed in the Statistical Package for the Social Sciences software (SPSS Inc., Chicago, IL, USA). Parameters were compared using two-way analysis of variance (ANOVA) followed by a Tukey’s post hoc test for multiple comparisons. Two-way repeated-measures ANOVA and Tukey’s post hoc tests were used for BP analysis.

## 5. Conclusions

In conclusion, high-fructose diet and minocycline treatment in pregnancy and lactation, both individually and together, lead to hypertension in adult male offspring. Although both insults induce similar degrees of high BP, there is no synergistic effect between them, and programming processes are driven by both overlapping and differential mechanisms. Our data highlight a close link among gut microbiome, SCFAs and their receptors, and RAS in hypertension of developmental origins programmed by HF and minocycline exposure. With a better understanding of the interplay among gut microbiota, SCFAs, and RAS that underlies HF- and minocycline-induced programmed hypertension, our results may aid in developing effective early interventions to prevent hypertension programmed by maternal excessive fructose consumption and antibiotic exposure.

## Figures and Tables

**Table 1:** Morphological and biochemical values.

Groups	ND	HF	NDM	HFM	*p*-Value		
Number	*N* = 8	*N* = 8	*N* = 8	*N* = 8	HF	M	HF × M
Mortality	0%	0%	0%	37.5%	-	-	-
Body weight (BW) (g)	350 ± 9	417 ± 15 *	343 ± 9	350 ± 8 #	0.004	0.004	NS
Left kidney weight (g)	1.59 ± 0.066	1.89 ± 0.089 *	1.65 ± 0.067	1.73 ± 0.05	0.018	NS	NS
Left kidney weight/100 g BW	0.45 ± 0.012	0.45 ± 0.015	0.48 ± 0.011 *	0.50 ± 0.013 #	NS	0.016	NS
Systolic BP (mmHg)	134 ± 1	142 ± 1 *	140 ± 1 *	145 ± 1 *	<0.001	<0.001	NS
Diastolic BP (mmHg)	90 ± 2	94 ± 1	99 ± 1 *	97 ± 2 *	NS	0.002	NS
MAP (mmHg)	105 ± 1	110 ± 1 *	113 ± 1 *	113 ± 2 *	0.046	<0.001	NS
Creatinine (µM)	19.5 ± 0.7	20.8 ± 2.4	17.1 ± 0.2 *	14.2 ± 0.3 #	NS	0.002	NS
CCr (mL/min/Kg BW)	2.77 ± 0.17	3.31 ± 0.39	3.85 ± 0.37 *	4.46 ± 0.16 #	NS	0.001	NS

*N* = 8/group; BP = blood pressure; MAP = mean arterial pressure; CCr = clearance of creatinine; ND = control rat received normal diet; HF = rats received high-fructose diet; NDM = control rat received normal diet and minocycline administration; HFM = rats received high-fructose diet and minocycline administration. * *p* < 0.05 vs. ND; # *p* < 0.05 vs. HF. HF × M = the interaction between high-fructose diet and minocycline.

**Table 2:** Plasma and fecal short chain fatty acid (SCFA) levels.

Groups	ND	HF	NDM	HFM	*p*-Value		
Feces, mM/g Feces					HF	M	HF × M
Acetic acid	4.53 ± 0.43	4.02 ± 0.39	3.89 ± 0.43	3.60 ± 0.19	NS	NS	NS
Propionic acid	1.38 ± 0.34	1.11 ± 0.13	1.74 ± 0.15	1.09 ± 0.14	NS	NS	NS
Isobutyric acid	0.17 ± 0.03	0.27 ± 0.03 *	0.18 ± 0.01	0.30 ± 0.04 †	0.001	NS	NS
Butyric acid	2.06 ± 0.22	1.45 ± 0.23	1.43 ± 0.19	1.69 ± 0.30	NS	NS	NS
Isovaleric acid	0.13 ± 0.03	0.30 ± 0.04 *	0.16 ± 0.01	0.38 ± 0.08 †	<0.001	NS	NS
Valeric acid	0.16 ± 0.03	0.20 ± 0.02	0.14 ± 0.01	0.23 ± 0.03 †	0.014	NS	NS
Plasma, µM					HF	M	HF × M
Acetic acid	195 ± 13	302 ± 27 *	209 ± 14	198 ± 16 #	0.019	0.029	0.006
Propionic acid	1.48 ± 0.20	1.46 ± 0.37	1.79 ± 0.17	1.32 ± 0.34	NS	NS	NS
Isobutyric acid	0.55 ± 0.15	ND	ND	0.36 ± 0.10	NS	NS	0.031
Butyric acid	2.13 ± 0.43	1.92 ± 0.29	1.25 ± 0.16 *	1.25 ± 0.42	NS	0.032	NS
Isovaleric acid	1.06 ± 0.42	0.31 ± 0.16	0.31 ± 0.06	0.69 ± 0.29	NS	NS	0.049
Valeric acid	6.07 ± 0.40	3.52 ± 0.79	3.11 ± 0.58	5.48 ± 0.55	NS	NS	0.001

*N* = 5–8/group; ND = not detectable; NS = no significance; * *p* < 0.05 vs. ND; † *p* < 0.05 vs. NDM; NS = not significant; HF × M = the interaction between high-fructose diet and minocycline.

**Table 3:** Nitric oxide (NO)-related parameters.

Groups	ND	HF	NDM	HFM	*p*-Value		
Plasma					HF	M	HF × M
l-citrulline (µM)	51.6 ± 5.4	50.3 ± 2	75.7 ± 5.5 *	51.6 ± 3.1 †	NS	0.011	0.021
l-arginine (µM)	199 ± 12	202 ± 12	266 ± 17 *	159 ± 6 †	0.001	NS	<0.001
ADMA (µM)	1.3 ± 0.1	1.5 ± 0.2	1.6 ± 0.2	1.3 ± 0.2	NS	NS	NS
SDMA (µM)	2 ± 0.2	1.8 ± 0.2	1.8 ± 0.2	1.9 ± 0.4	NS	NS	NS
l-arginine-to-ADMA ratio (µM/µM)	154 ± 11	170 ± 33	198 ± 42	134 ± 24	NS	NS	NS

*N* = 5–8/group; ADMA = asymmetric dimethylarginine; SDMA = symmetric dimethylarginine; * *p* < 0.05 vs. ND; † *p* < 0.05 vs. NDM.

**Table 4:** PCR primer sequences.

Gene	Forward	Reverse
GPR41	5 tcttcaccaccgtctatctcac 3	5 cacaagtcctgccaccctc 3
GPR43	5 ctgcctgggatcgtctgtg 3	5 cataccctcggccttctgg 3
GPR91	5 gtcgtctgggccttagtgacc 3	5 gctgccttctgattcatgtgg 3
Olfr78	5 gaggaagctcacttttggtttgg 3	5 cagcttcaatgtccttgtcacag 3
Renin	5 aacattaccagggcaactttcact 3	5 acccccttcatggtgatctg 3
PRR	5 gaggcagtgaccctcaacat 3	5 ccctcctcacacaacaaggt 3
AGT	5 gcccaggtcgcgatgat 3	5 tgtacaagatgctgagtgaggcaa 3
ACE	5 caccggcaaggtctgctt 3	5 cttggcatagtttcgtgaggaa 3
AT1R	5 gctgggcaacgagtttgtct 3	5 cagtccttcagctggatcttca 3
R18S	5 gccgcggtaattccagctcca 3	5 cccgcccgctcccaagatc 3

GPR41 = G protein-coupled receptor 41; GPR43 = G protein-coupled receptor 43; GPR91 = G protein-coupled receptor 91; Oflr78 = olfactory receptor 78; PRR = prorenin receptor; AGT = angiotensinogen; ACE = angiotensin converting enzyme; AT1R = angiotensin II type 1 receptor; R18S = 18S ribosomal RNA.

## Data Availability

Data will be available upon request.
